# Reliability of circulating fibrinogen in the diagnosis of prosthesis-related infections: a systematic review and meta-analysis

**DOI:** 10.1186/s13018-020-02171-x

**Published:** 2021-01-09

**Authors:** Xingyang Zhu, Haitao Zhang, Xiaobo Sun, Yijin Li, Jiahao Li, Yuqing Zeng, Peng Deng, Xinyu Qi, Jinlun Chen, Pengcheng Ye, Wenjun Feng, Jie Li, Jianchun Zeng, Yirong Zeng

**Affiliations:** 1 The First Clinical Medical School, Guangzhou University of Chinese Medicine, Jichang Road 12#, District Baiyun, Guangzhou, Guangdong China; 2 Yichuan People’s Hospital, Jiuchang Road 21#, District Yichuan, Luoyang, Henan China; 3 Department of Orthopaedics, The First Affiliated Hospital of Guangzhou University of Chinese Medicine, Jichang Road 16#, District Baiyun, Guangzhou, 510405 Guangdong China

**Keywords:** Periprosthetic joint infection, Fibrinogen, Diagnosis, Joint arthroplasty

## Abstract

**Background:**

Fibrinogen (FIB) has recently been used as a biomarker to diagnose periprosthetic joint infection (PJI), but its reliability is still questionable. The aim of this study was to investigate the accuracy of FIB in the diagnosis of PJI after joint replacement.

**Methods:**

We searched for literatures published in PubMed, EMBASE, and the Cochrane Library from the time of database inception to September 2020 and screened the studies according to the inclusion criteria. Then, we calculated the diagnostic parameters of FIB, including the pooled sensitivity, specificity, positive likelihood ratio (PLR), negative likelihood ratio (NLR), area under the curve (AUC), and diagnostic odds ratio (DOR). In addition, we implemented subgroup analyses to identify the sources of heterogeneity.

**Results:**

Seven studies including 1341 patients were selected in our meta-analysis. The pooled sensitivity, specificity, PLR, NLR, and DOR of FIB for PJI diagnosis were 0.78 (95% confidence interval [CI], 0.73–0.82), 0.83 (95% CI, 0.81–0.86), 4.60 (95% CI, 3.30–6.42), 0.24 (95% CI, 0.18–0.34), and 20.13 (95% CI, 14.80–27.36), respectively, while the AUC was 0.896.

**Conclusion:**

The present study indicated that FIB was a reliable detection method and might be introduced into the diagnostic criteria for PJI. However, more robust studies are still needed to confirm the current findings, because most of the included studies were retrospective and had small sample sizes.

**Supplementary Information:**

The online version contains supplementary material available at 10.1186/s13018-020-02171-x.

## Introduction

Periprosthetic joint infection (PJI) is a frustrating complication after joint arthroplasty. It is well known that the success of any treatment regimen depends largely on the time of early diagnosis [1], and so is the diagnosis of PJI. If not diagnosed promptly and correctly, it will lead to devastating consequences [2, 3]. In addition, its cost is 4–5 times more than that of primary arthroplasty, due to multiple operations, prolonged recovery time, and long-term use of antibiotics and analgesics [4, 5]. Natheless, many patients with PJI are often treated according to the principle of aseptic loosening, resulting in a significantly higher failure rate, due to the high false-negative rate of bacterial culture of both synovial fluid and incision secretion [6].

Although PJI has a series of diagnostic criteria currently, such as the American Academy of Orthopedic Surgeon (AAOS) [7], Musculoskeletal Infection Society (MSIS) [8], Infectious Diseases Society of America (IDSA) [9], International Consensus Meeting (ICM) [10], and a new definition of 2018 [11], so far, PJI with a low toxicity or negative culture may still easily be missed [12, 13]. Therefore, new diagnostic methods still need to be found to further improve the accuracy of PJI diagnosis.

In recent years, the role of biomarkers, such as d-dimer, fibrin degradation products, α-defensin, leukocyte esterase, and interleukin-6 of plasma or synovial fluid in the diagnosis of PJI, has been reported; however, their diagnostic value has been unsatisfactory due to either poor sensitivity or specificity [14–16]. Therefore, to date, the diagnosis of PJI has not been effectively confirmed by the application of a single biomarker.

However, some scholars recently have reported the good performance of circulating fibrinogen (FIB) for diagnosing PJI, and believe that its diagnostic value should be higher than d-dimer, and it could be comparable to the traditional biomarkers such as C-reactive protein (CRP) and erythrocyte sedimentation rate (ESR) [17, 18]. Nevertheless, their conclusions have been questioned because of the small sample size and variable results of these studies. Therefore, the purpose of this systematic review and meta-analysis was to evaluate the performance of FIB for diagnosing PJI.

## Methods

This current systematic review and meta-analysis was performed in accordance with the Preferred Reporting Items for Systematic Reviews and Meta-Analyses (PRISMA) [19]. The research protocol had not been registered, and ethical approval was not required, because this study only involved a review of published literature, without involving the new patient data. Before starting the literature search, all coauthors agreed to the protocol.

### Search strategy

Following the PICOS methodology, two authors (Xingyang Zhu and Haitao Zhang) developed the search strategy with the assistance of an experienced librarian. A comprehensive search of all relevant studies up to September 24, 2020, was carried out through PubMed, EMBASE, and Cochrane Collaboration Library. The Medical Subject Headings and entry terms contained in the search strategy were as follows: “Prosthesis-Related Infections” OR “Prosthesis Related Infections” OR “Infections, Prosthesis-Related” OR “Prosthesis-Related Infection” OR “Peri-Prosthetic Joint Infection” OR “Periprosthetic Joint Infection” OR “Prosthetic joint infection” OR “PJI” standed for disease, “Fibrinogen” OR “Blood Coagulation Factor I” OR “Coagulation Factor I” OR “Factor I, Coagulation” OR “Factor I” OR “gamma-Fibrinogen” OR “gamma Fibrinogen” represented target index. The language was limited to English. In addition, a manual search of possibly relevant bibliographies was also conducted for additional citations. The detailed search strategy is shown in Additional file 1.

### Selection criteria

The inclusion criteria of the literature were as follows: (1) focused on the value of FIB in the diagnosis of PJI; (2) directly or indirectly provided the following data: true positive, false negative, false positive, and true negative; and (3) diagnosed PJI based on widely recognized gold standards, such as MSIS or ICM.

The exclusion criteria mainly included the following: (1) animal studies, (2) studies that were incompleteness of data, (3) reduplicative studies of the same cases in different periods, and (4) reviews, case reports, and commentaries.

Two reviewers independently scanned the titles, abstracts, and full texts and selected the literatures based on the eligibility criteria. If they encountered any divergences, they could reach an agreement through discussion or seek help from professor Yirong Zeng.

### Quality assessment

The Quality Assessment of Diagnostic Accuracy Studies-2 (QUADAS-2) [20], which is composed of patient selection, index testing, reference standard, and flow and timing, was used to evaluate the quality of each included study in the Revman software (version 5.3). Two reviewers evaluated independently the quality of eligible studies, and in the event of any divergences, the third author decided the final result.

### Data extraction

Relevant information was extracted by two reviewers independently from all selected studies with a standardized data collection form, which included the following variables: author, year of publication, study type, average age, sex, body mass index (BMI), number of participants, detection method, sample type, level range, and diagnostic criteria. The interest outcomes of our study included threshold value, true positive (TP), false positive (FP), true negative (FN), false negative (TN), and area under the curve (AUC). The third author resolved any discrepancies that arose during this process.

### Statistical analysis

All statistical analyses were done with the MetaDiSc (1.4) or Stata software (14.0), and *P* value < 0.05 was considered to be statistically significant. The extracted raw data were used to calculate the pooled sensitivity, specificity, positive likelihood ratio (PLR), negative likelihood ratio (NLR), diagnostic odds ratio (DOR), and AUC. The *I*^2^ statistics were performed to estimate the heterogeneity across studies. If the heterogeneity test expressed *I*^2^ < 50%, data were pooled by a fixed-effects model, while the random effects were suitable for significant heterogeneity (*I*^2^ ≥ 50%). Forest plots were applied to depict the results of each study and to evaluate pooled estimates, while Deeks’ funnel plots were used to assess publication bias. Meta-regression and subgroup analysis were conducted to explore the potential sources of heterogeneity if it was necessary, and sensitivity analysis was conducted to determine the stability of the outcomes.

## Results

### Study selection

**Fig. 1:**
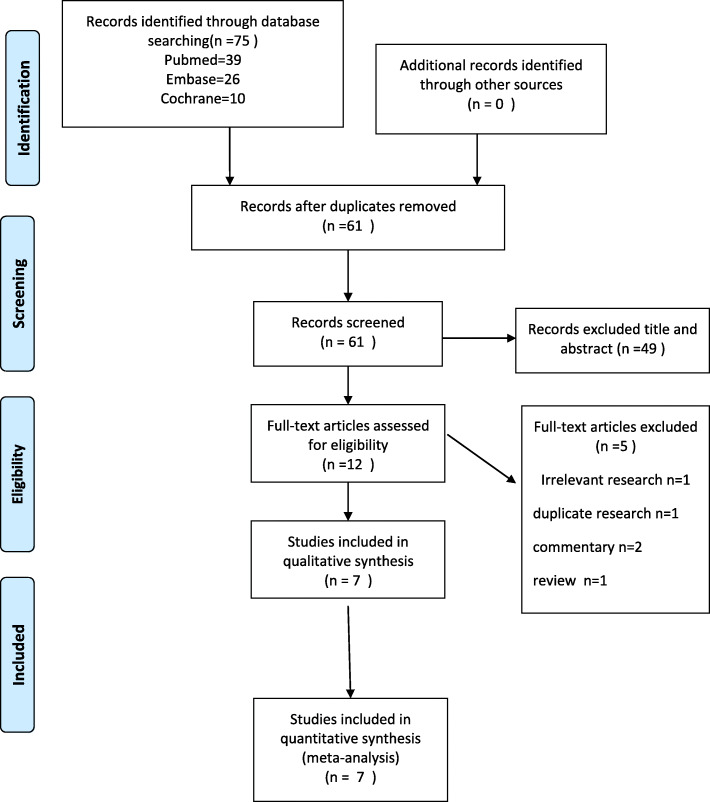
Summary of the evidence search and selection process

Through initial searches of 3 databases, 75 articles were selected. Fourteen duplicates were deleted, leaving 61 articles for screening. Forty-nine irrelevant citations were excluded after screening the titles and abstracts, leaving 12 papers for review. Five studies were rejected for several reasons, such as irrelevant research (*n* = 1), duplicate research (*n* = 1), commentary (*n* = 2), or review (*n* = 1). Finally, 7 references were included in this study [17, 18, 21–25]. The details of the study selection process can be found in Fig. 1.

### Quality assessment

**Fig. 2:**
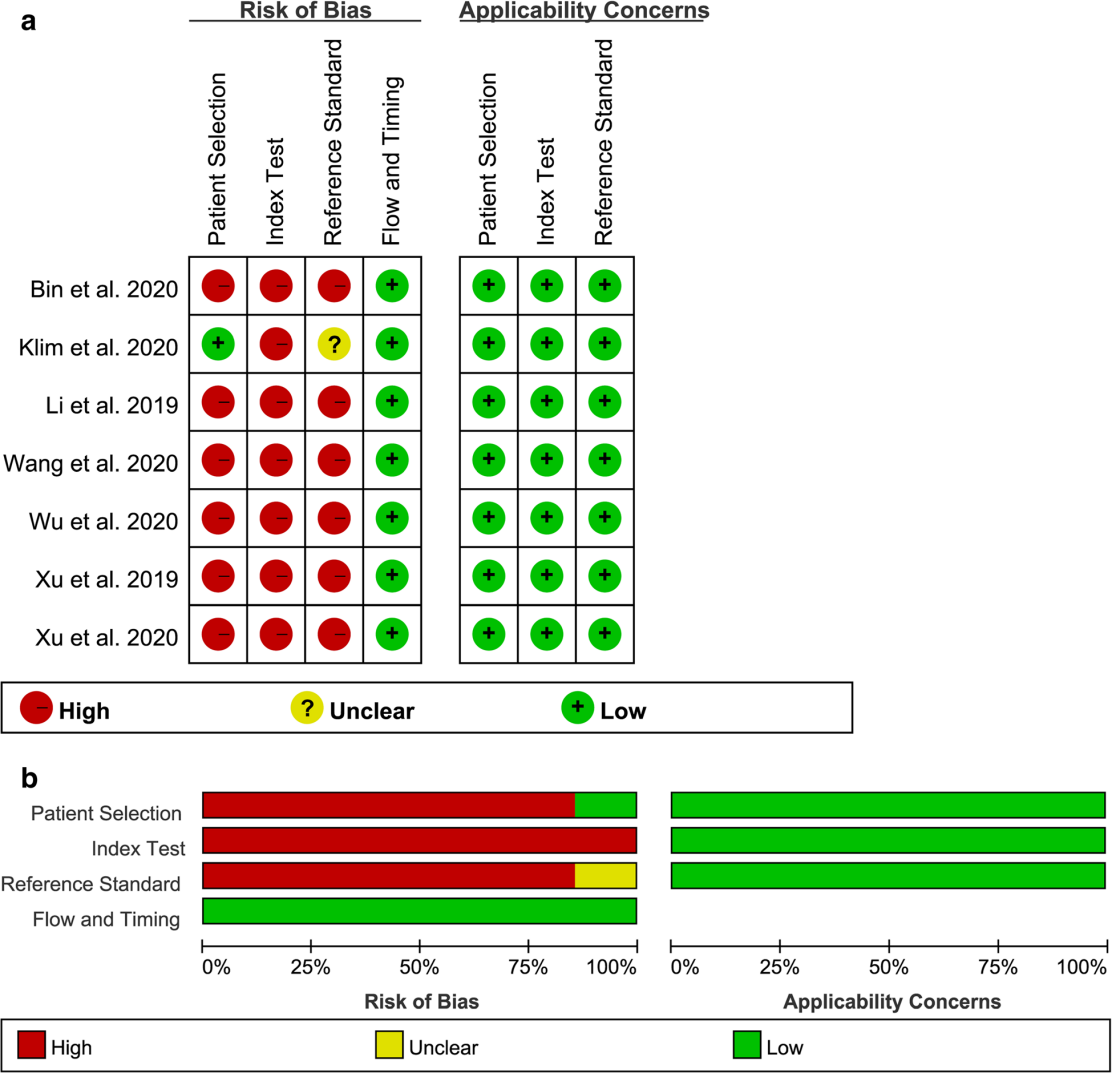
**a**, **b** The quality assessment for 7 studies

According to the QUADAS-2 tool, the quality assessment for 7 studies is shown in Fig. 2. As shown in the figure, the risks of bias for clinical applicability were low in all studies, so was the flow and timing. However, the patient selection and reference standard were both high risk, because 6 studies did not avoid case-control designs and interpretations of reference standards were not blinded [17, 18, 21–23, 25]. In addition, the thresholds of FIB for the 7 studies were not pre-specified; therefore, all index texts were high risk [17, 18, 21–25].

### Study characteristics

Six retrospective [17, 18, 21–23, 25] and one prospective [24] case-control studies, including 1341 patients, were finally selected. All studies were single-center [17, 18, 22–25] except for one multicenter study [21]. Among the 7 studies, 6 were from China [17, 18, 21–23, 25] and 1 from the USA [24]. All 7 studies, including PJI involving knee or hip joints, were published in the past 2 years. In addition, FIB was derived from the serum in 2 studies [23, 24] and from the plasma in the remaining 5 studies [17, 18, 21, 22, 25]. Three studies provided the detection methods of FIB [21, 24, 25], while the remaining studies did not mention it. Six studies used ICM as the “gold standard” for diagnosing PJI [17, 18, 21–23, 25], while only one adopted MSIS as the reference standard [24]. The diagnostic thresholds of FIB were not predetermined but were obtained from the receiver operator characteristic curve (ROC) in all 7 studies. The main features and related results of the included studies are shown in Tables 1 and 2.

**Table 1:** Characteristics of the included studies

Author	Study type	Mean age (range, years)		Gender		BMI		Participants		Detection method	Level range (N/PJI)		Threshold value	Gold standard
		N-PJI	PJI	F	M	N-PJI	PJI	N-PJI	PJI		N-PJI	PJI		
Xu et al. 2020 [25]	R	NV	NV	NV	NV	NV	NV	207	153	NV	2.96 ± 0.79	4.18 ± 1.16	3.57 g/L	ICM
Klim et al. 2020 [24]	P	65.1 ± 14.6	65.7 ± 15.8	46	38	NV	NV	29	55	Coagulometry with sodium citrate blood	NV	NV	515 mg/dL	MSIS
Xu et al. 2019 [16, 22]	R	NV	53.3 ± 14.9	56	46	25.3 ± 3.5	24.6 ± 4.0	94	8	STA-R Evolution analyzer	NV	NV	3.61 g/L	ICM
Wu et al. 2020 [18]	R	69.13 ± 11.19	62.64 ± 11.58	68	41	24.49 ± 5.87	24.75 ± 3.90	76	33	NV	3.01 ± 0.72	4.81 ± 1.87	3.61 g/L	ICM
Li et al. 2019 [21]	R, M	61.3 (23–86)	63.7 (18–89)	NV	NV	25.15 (14.93–46.66)	25.01 (16.71–33.06)	363	76	STA-R Evolution analyzer or Sysmex CS-5100 System	NV	NV	4.01 g/L	ICM
Wang et al. 2020 [17, 27]	R	63.4 (23–87)	64.6 (37–82)	87	70	NV	NV	106	51	NV	NV	NV	3.56 g/L	ICM
Bin et al. 2020 [23]	R	60.30 ± 13.79	62.13 ± 11.37	52	38	23.62 ± 3.67	23.72 ± 4.26	37	53	NV	2.86 (2.46–3.25)	4.37 (3.82–4.95)	3.60 g/L	ICM

*PJI* peri-prosthetic joint infection, *N-PJI* not periprosthetic joint infection, *BMI* body mass index, *P* prospective study, *R* retrospective study, *M* multicenter study, *F* female, *M* male, *NA* not applicable, *MSIS* Musculoskeletal Infection Society, *ICM* Internal Consensus Meeting

**Table 2:** Data extracted for the construction of 2 × 2 table

Author	Year	TP	FP	FN	TN
Xu et al. [25]	2020	105	29	48	178
Klim et al. [24]	2020	52	8	3	21
Xu et al. [16, 22]	2019	7	35	1	59
Wu et al. [18]	2020	25	10	8	66
Li et al. [21]	2019	58	50	18	313
Wang et al. [17, 27]	2020	44	17	7	89
Bin et al.	2020	42	2	11	35

*TP* true positive, *FP* false positive, *FN* false negative, *TN* true negative

### Diagnostic accuracy

**Fig. 3:**
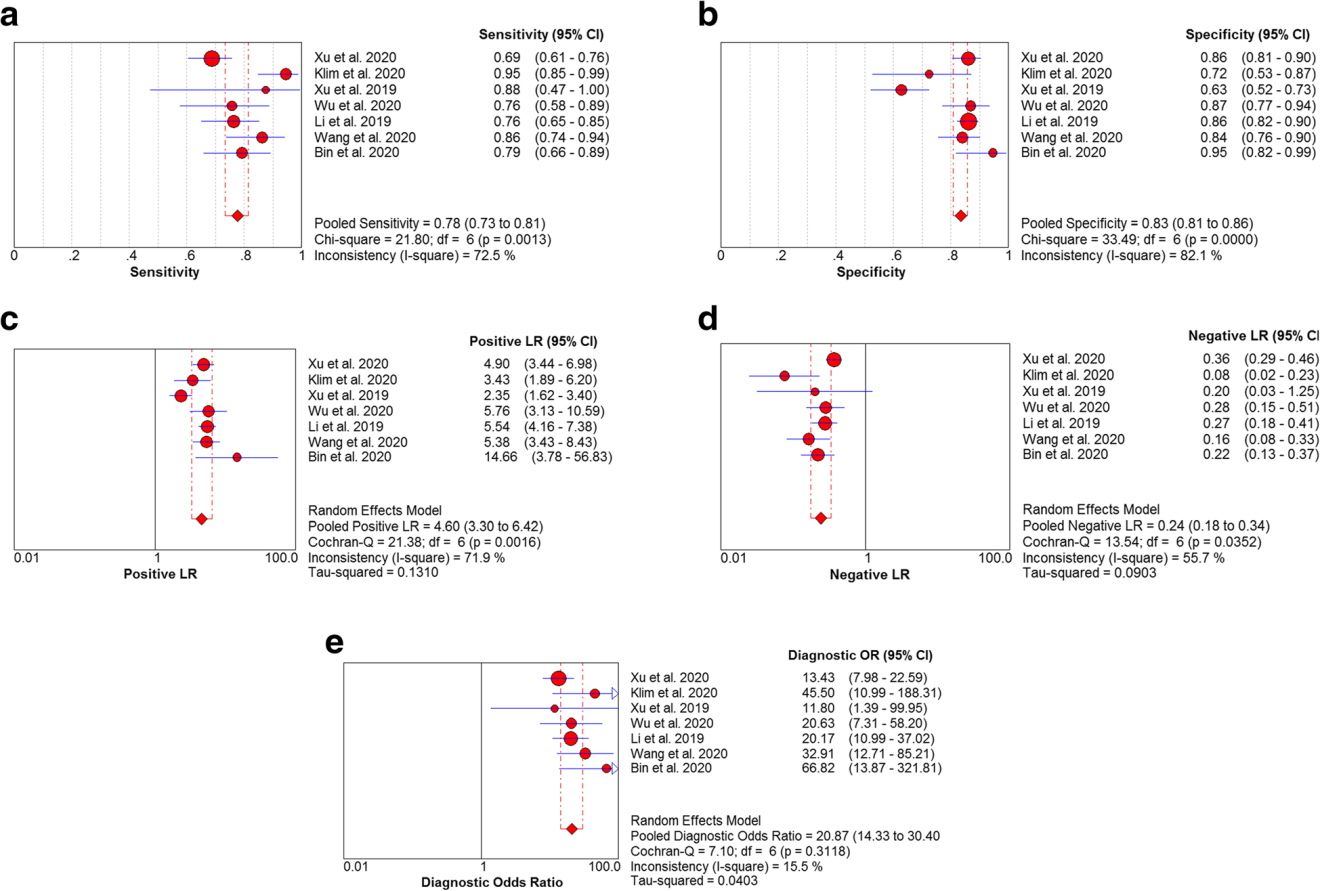
The pooled sensitivity and specificity of FIB for PJI diagnosis (**a**, **b**). The pooled PLR, NLR, and DOR (**c**–**e**)

**Fig. 4:**
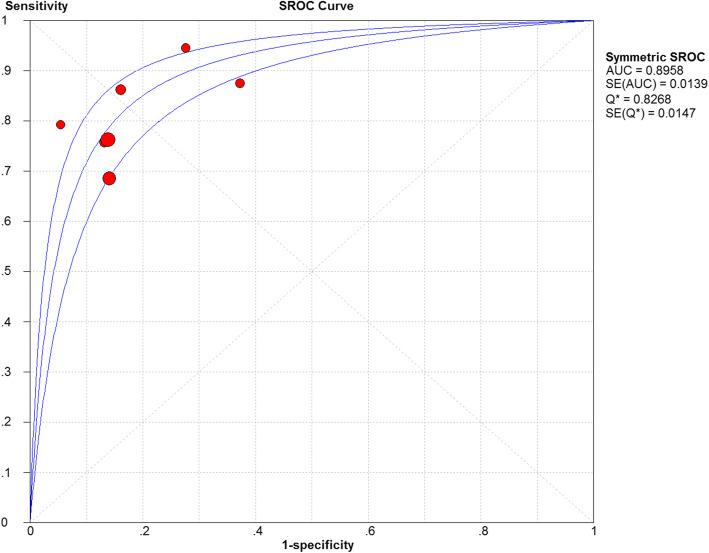
SROC curve

Due to the significant heterogeneity in the sensitivity (*I*^2^ = 72.5%, *P* > 0.001), specificity (*I*^2^ = 82.1, *P* < 0.001), PLR (*I*^2^ = 71.9, *P* > 0.001), and NLR (*I*^2^ = 55.7, *P* > 0.001), the random effects model was adopted. The pooled sensitivity and specificity of FIB for PJI diagnosis were 0.78 (95% CI, 0.73–0.82) and 0.83 (95% CI, 0.81–0.86), respectively (Fig. 3a, b). The pooled PLR, NLR, and DOR were 4.60 (95% CI, 3.30–6.42), 0.24 (95% CI, 0.18–0.34), and 20.13 (95% CI, 14.80–27.36), respectively (Fig. 3c–e), and the AUC was 0.896 (Fig. 4).

### Heterogeneity analysis

**Fig. 5:**
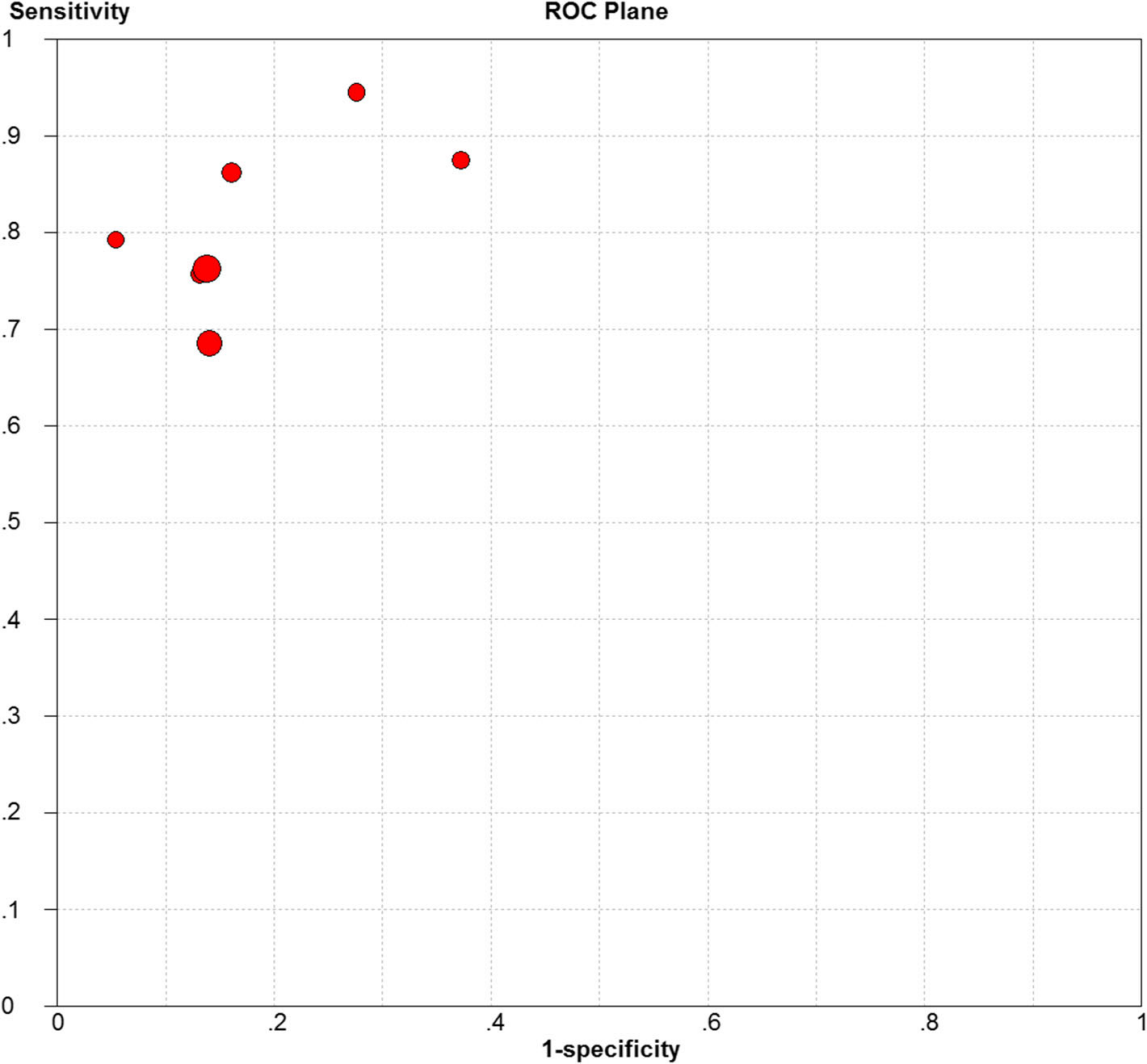
ROC plane

The Spearman correlation coefficient was 0.643 (*P* = 0.119), indicating that the heterogeneity might be independent of the threshold effect. Meanwhile, the Cochran *Q* test of DOR obtained Cochran *Q* = 7.10 (*P* = 0.3118), suggesting that the heterogeneity of this study was related to the non-threshold effects. The above result could also be obtained from the no shoulder-like ROC plane (Fig. 5).

### Subgroup analysis

The subgroup results of plasma FIB and studies from China are presented in Table 3. In the subgroup of plasma FIB [17, 18, 21, 22, 25], the pooled sensitivity and specificity were 0.74 (95% CI, 0.69–0.79) and 0.83 (95% CI, 0.81–0.86), respectively, while the pooled sensitivity and specificity were 0.75 (95% CI, 0.70–0.79) and 0.84 (95% CI, 0.81–0.86), respectively, in the subgroup of studies from China [17, 18, 21–23, 25].

**Table 3:** Subgroup analysis of FIB for PJI diagnosis

Subgroup	No. of studies	Sensitivity (95% CI)	Specificity (95% CI)	PLR (95% CI)	NLR (95% CI)	DOR (95% CI)	AUC
Plasma FIB	5	0.74 (0.69–0.79)	0.83 (0.81–0.86)	4.51 (3.14–6.47)	0.29 (0.22–0.38)	17.85 (12.71–25.07)	0.8945
China	6	0.75 (0.70–0.79)	0.84 (0.81–0.86)	4.84 (3.31–7.09)	0.28 (0.21–0.36)	19.56 (13.46–28.43)	0.8880

*FIB* fibrinogen, *CI* confidence interval, *PLR* positive likelihood ratio, *NLR* negative likelihood ratio, *DOR* diagnostic odds ratio, *AUC* area under the curve

### Sensitivity analysis

**Fig. 6:**
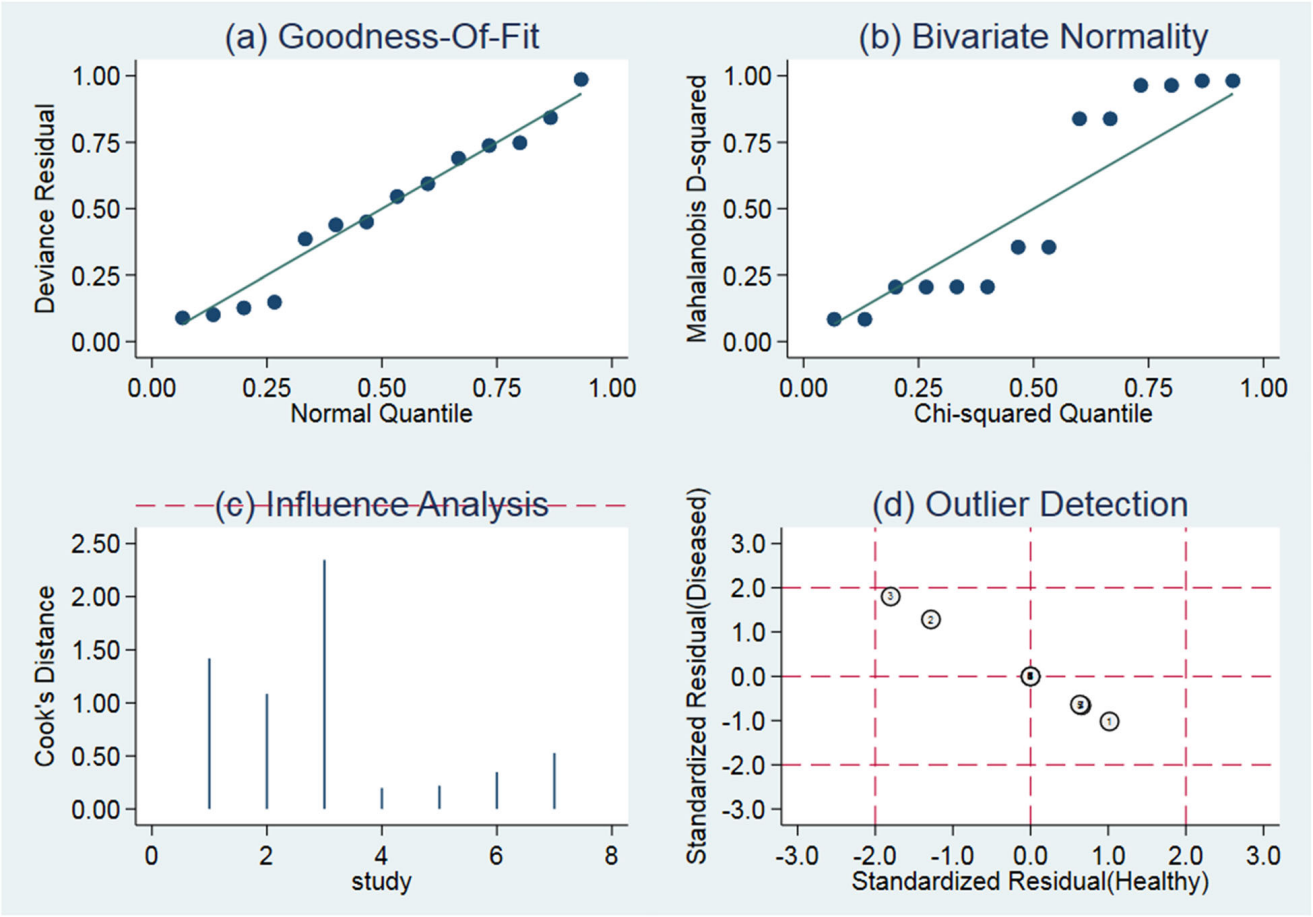
**a** Goodness-of-fit. **b** Bivariate normality. **c** Influence analysis. **d** Outlier detection

Figure 6 demonstrates that all the included studies would not cause sensitivity to the combined results. Therefore, the results of this study should be relatively stable.

### Publication biases

**Fig. 7:**
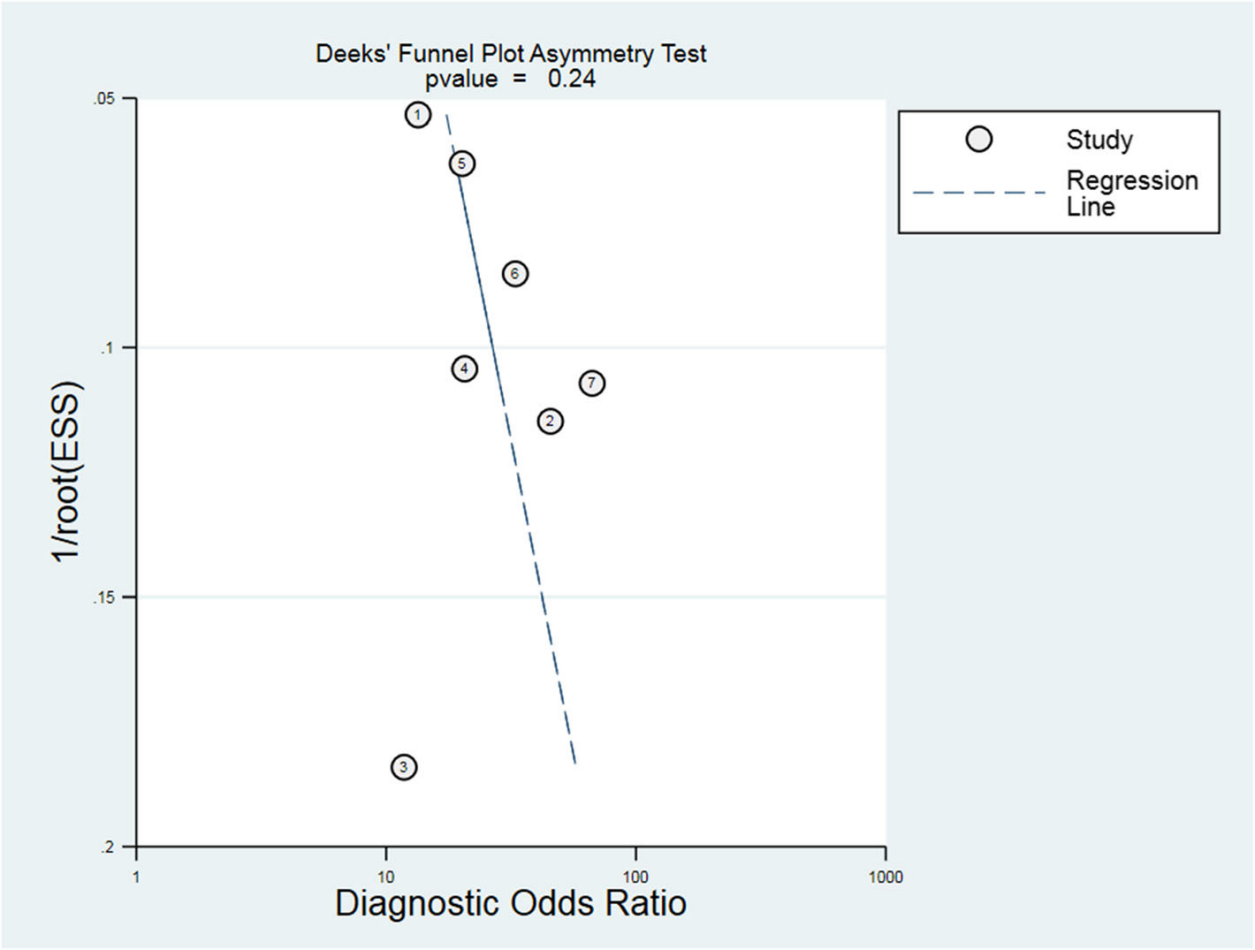
Deeks’ funnel plot asymmetry test

As shown in Fig. 7, Deeks’ funnel plot asymmetry test has a *P* value of 0.24, indicating that publication bias might not exist.

## Discussion

Both d-dimer and FIB are important biomarkers of the coagulation system. d-dimer was once considered to have good performance in the diagnosis of PJI [26, 27]; however, more and more recent studies have demonstrated that d-dimer has limited value in PJI diagnosis [15, 16, 28–30]. For example, compared with 26 patients with aseptic loosening, Huang et al. [15] found that there was no significant difference in the level of plasma d-dimer in 31 patients with PJI. Another retrospective study on 318 patients (129 PJI and 189 aseptic mechanical failure) conducted by Xu et al. [16] showed that the sensitivity and specificity of d-dimer in PJI diagnosis were only 68.29% and 50.70%, respectively, which were significantly inferior to traditional biomarkers, such as ESR and CRP. In addition, the d-dimer test results from different laboratories vary greatly, as laboratories may use different testing methods due to the lack of standardization, which may lead to very different results for testing the same sample. For example, Pearson et al. [28] proved the variability of d-dimer results through test data from 3903 laboratories. Meanwhile, the authors used the cutoff value recommended by ICM to estimate the classification of patients, and the results showed that many patients were misclassified clinically [28]. Therefore, the general cutoff value of d-dimer is not appropriate in the diagnostic criteria of PJI.

FIB, as another important blood coagulation marker, has been shown to be closely related to inflammation-associated pathology [31–34]. In fact, the inflammation/infection mechanism and the coagulation cascade are inseparable processes. For example, systemic or local infection can lead to systemic coagulation abnormalities, increased fibrinolytic activity, and circulating FIB concentration; conversely, coagulation abnormalities sometimes also indicate the presence of systemic or local infections [31–33]. In the previous literature, FIB had been reported to predict or evaluate the progress of inflammatory diseases such as appendicitis [35, 36], periodontitis [37, 38], malaria [39], and sepsis [40]. But, it was not until 2018 that Klim et al. [41] first reported the role of FIB in diagnosing PJI. Then, several studies evaluated the diagnostic value of FIB in PJI by comparing it with CRP, ESR, white blood cell count (WBC), or d-dimer [17, 18, 21]. In addition, fortunately, the latest researches have shown that FIB, as an upstream product of d-dimer, has better diagnostic performance than d-dimer [17, 18, 21]. For example, a study by Wu et al [18] showed that the sensitivity, specificity, DOR, and AUC of FIB in the diagnosis of PJI were significantly better than those of d-dimer (75.8% vs 75.8%, 86.4% vs 67.0%, 64.1% vs 42.4%, 91.8% vs 89.6%, respectively). Another study also revealed that FIB showed superior performance than d-dimer in PJI diagnosis, and its value was comparable to CRP and ESR [21]. However, due to limited original research, we were unable to simultaneously compare the diagnostic value of FIB with CRP, ESR, and d-dimer by combining the effect sizes in this meta-analysis.

It is well known that malignancy, thrombosis, cardiovascular diseases, cerebrovascular diseases, autoimmune diseases, and systemic infectious diseases all contribute to the increase of plasma FIB. Therefore, FIB has poor diagnostic accuracy for PJI in patients with these diseases [18], while traditional markers such as ESR and CRP also have the same shortcomings. Xu et al. [25] evaluated 79 patients with coagulation-related comorbidities and found that the sensitivity and specificity of FIB for PJI diagnosis were only 76.7% and 72.2%, respectively. Therefore, the authors suggested that FIB may only be used as an auxiliary diagnostic method in this population. Another study showed that the diagnostic accuracy of plasma FIB in the malignant subgroup was significantly better than that of autoimmune diseases and cardiovascular and cerebrovascular diseases [21]. However, in this meta-analysis, such patients were excluded or analyzed separately in the included studies, which would inevitably improve the diagnostic efficiency of FIB and restrict the generalization of our conclusions.

Since no consensus has been reached on the use of a single threshold so far, different cutoff values of FIB, ranging from 3.56 to 5.15 g/L, were applied in the diagnosis of PJI in the present study. Similar to d-dimer, the diagnostic threshold of FIB may be different in patients with coagulation-related diseases or systemic inflammatory diseases compared with patients without comorbidities [25]. In addition, the diagnostic threshold for PJI involving the knee or hip may also be different [18]. Therefore, the appropriate cutoff value of FIB in the diagnosis of PJI still needs to be studied.

Two previous meta-analyses have shown that plasma d-dimer is more valuable in the diagnosis of PJI than serum d-dimer [29, 30]. In our study, plasma FIB and serum FIB were also included. However, due to the limited literature involving serum FIB, it is difficult to conduct a subgroup analysis to compare the differences between them. In addition, different detection methods of FIB were used in the included studies, which inevitably caused heterogeneity between studies.

On the whole, our pooled data showed that circulating FIB would be an excellent biomarker for diagnosing PJI with an AUC of 0.896, corresponding to a sensitivity of 78% and a specificity of 83%. It is well known that LR and DOR have been generally used to demonstrate the validity of diagnostic indicators [42]. Based on this meta-analysis, the pooled PLR, NLR, and DOR were 4.60, 0.24, and 20.13, respectively. A principle defines PLR > 2, NLR < 0.5, or DOR > 4 is considered to be a viable predictor, while PLR > 5, NLR < 0.2, or DOR > 10 is considered to be a good predictor [43]. Therefore, as far as LR is concerned, FIB is a feasible indicator for PJI diagnosis and may be a good predictive parameter when the DOR is used as a reference. In addition, FIB is a routine examination performed by hospitalized patients and will not increase their additional burden.

To the best of our knowledge, this appears to be the first meta-analysis to assess the accuracy of FIB in the diagnosis of PJI after a review of the literature. We noted that an excellent meta-analysis recently was published by Zhang et al. [44], but the purpose was mainly to compare the diagnostic efficacy of d-dimer and FIB for PJI, and only 3 articles in this meta-analysis were about FIB for diagnosing PJI, so the reliability of the results might be limited.

The present study does have some limitations. First, most of the included studies were retrospective case-control studies with small sample sizes, so the overall quality of this study was not high. Second, as mentioned above, in order to rule out other possible conditions related to elevated coagulation markers, patients with malignancy, thrombosis, liver diseases, or systemic inflammatory diseases were excluded from the included studies, inevitably leading to selective bias. Third, due to the incomplete original data, it was unable to calculate the optimal threshold of FIB, and it was difficult to perform a more detailed subgroup analysis. Fourth, due to the limited sensitivity of reference standard (MSIS or ICM), PJI might be missed, which might cause certain biases. Finally, these studies were completed in different regions or hospitals, and different test methods or sample sources were used, so there was significant heterogeneity between the studies.

## Conclusions

The present study indicated that FIB was an adequate test to diagnose PJI and would be introduced into the diagnostic criteria for PJI. However, most of the included studies were retrospective and had small sample sizes; therefore, our results should be interpreted with caution, and more robust studies are still needed to confirm the current findings.

## Supplementary Information

**Additional file 1.** Detailed search strategy.

## Data Availability

The authors declare that all the data supporting the findings of this study are available within the article and its supplementary information files.

## Abbreviations

PJI: Periprosthetic joint infection
FIB: Fibrinogen
THA: Total hip arthroplasty;
TKA: Total knee arthroplasty
MSIS: Musculoskeletal Infection Society
ICM: International Infection Consensus
TP: True positive
FP: False positive
FN: True negative
TN: False negative
PLR: Positive likelihood ratio
NLR: Negative likelihood ratio
DOR: Diagnostic odds ratio
AUC: Area under the curve
ROC: Receiver operator characteristic curve
CRP: C-reactive protein
ESR: Erythrocyte sedimentation rate
