# Supplementary material for: Reliability of circulating fibrinogen in the diagnosis of prosthesis-related infections: a systematic review and meta-analysis
Source: J Orthop Surg Res. 2021 Jan 9;16:31. doi: 10.1186/s13018-020-02171-x (PMC7797116; doi:10.1186/s13018-020-02171-x)

# 2020.09.24

# Pubmed

# #1 “Prosthesis-Related Infections” [Mesh] OR Prosthesis-Related Infections[Title/Abstract] OR Prosthesis Related Infections[Title/Abstract] OR Infections, Prosthesis-Related[Title/Abstract] OR Prosthesis-Related Infection[Title/Abstract] OR Peri-Prosthetic Joint Infection[Title/Abstract] OR Periprosthetic Joint Infection[Title/Abstract] OR Prosthetic joint infection[Title/Abstract] OR PJI[Title/Abstract]

# 13953

# #2 “Fibrinogen” [Mesh] OR Fibrinogen[Title/Abstract] OR Blood Coagulation Factor I[Title/Abstract] OR Coagulation Factor I[Title/Abstract] OR Factor I, Coagulation[Title/Abstract] OR Factor I[Title/Abstract] OR gamma-Fibrinogen[Title/Abstract] OR gamma Fibrinogen [Title/Abstract]

# 81324

# #3 #1 AND #2 39

**Embase**

**#1** ‘prosthesis Infection’/exp **9646**

# #2

# ‘Prosthesis-Related Infections’:ab,ti OR ‘Prosthesis Related Infections’:ab,ti OR ‘Infections, Prosthesis-Related’:ab,ti OR ‘Prosthesis-Related Infection’:ab,ti OR ‘Peri-Prosthetic Joint Infection’:ab,ti OR ‘Periprosthetic Joint Infection’:ab,ti OR ‘Prosthetic joint infection’:ab,ti OR ‘PJI’:ab,ti

# 3399

# #3 #1 OR #2 10513

**#4** ‘fibrinogen blood level’/exp  **9525**

**#5** ‘Fibrinogen’:ab,ti OR ‘Blood Coagulation Factor I’:ab,ti OR ‘Coagulation Factor I’:ab,ti OR ‘Factor I, Coagulation’:ab,ti OR ‘Factor I’:ab,ti OR ‘gamma-Fibrinogen’:ab,ti OR ‘gamma Fibrinogen’:ab,ti

**82766**

**#6 #4 OR #5 85533**

**#7 #3 AND #6 26**

**Cochrane**

**#1** MeSH descriptor: [Prosthesis-Related Infections] explode all trees

**#2** MeSH descriptor: [Fibrinogen] explode all trees

**#3**

(Prosthesis-Related Infections):ti,ab,kw OR (Prosthesis Related Infections):ti,ab,kw OR (Infections, Prosthesis-Related):ti,ab,kw OR (Prosthesis-Related Infection):ti,ab,kw OR (Peri-Prosthetic Joint Infection):ti,ab,kw OR (Periprosthetic Joint Infection):ti,ab,kw OR (Prosthetic joint infection):ti,ab,kw OR (PJI):ti,ab,kw

**#4**

(Fibrinogen):ti,ab,kw OR (Blood Coagulation Factor I):ti,ab,kw OR (Coagulation Factor I):ti,ab,kw OR (Factor I, Coagulation):ti,ab,kw OR (Factor I):ti,ab,kw OR (gamma-Fibrinogen):ti,ab,kw OR (gamma Fibrinogen):ti,ab,kw

**#5**  **#1 OR #3**

**#6** **#2 OR #4**

**#7** **#5 AND #6**


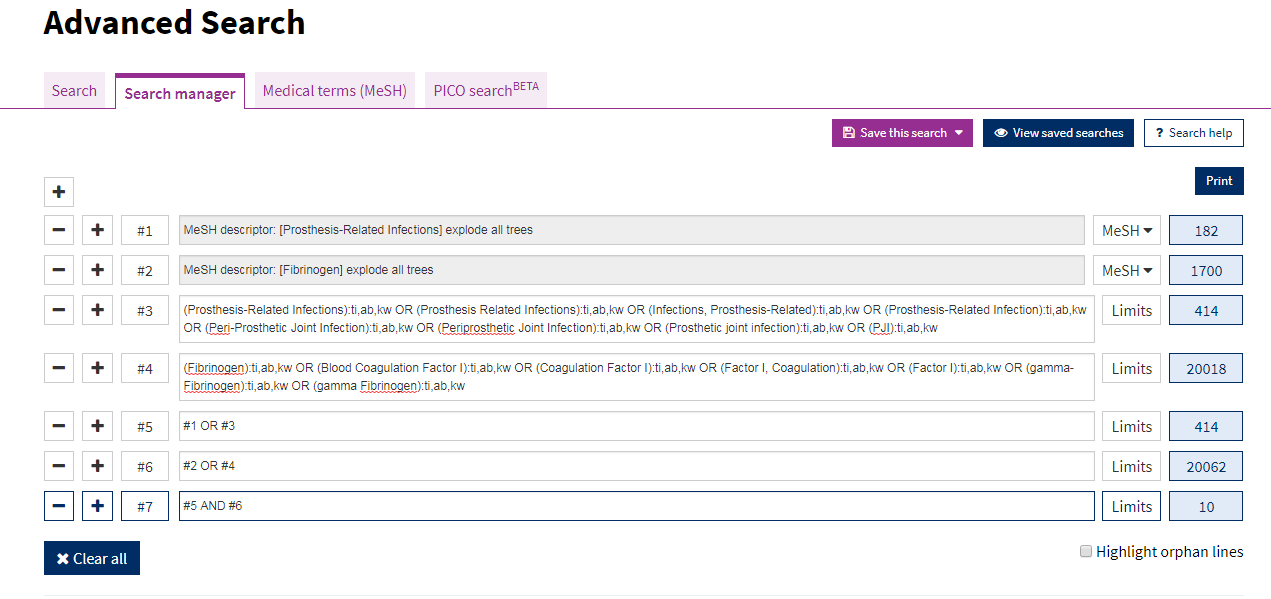

Supplement: Supplementary file 1 — Additional file 1. Detailed search strategy. [file 13018_2020_2171_MOESM1_ESM.docx]
